# Estimuladores de Guanilato Ciclase Solúvel (Riociguate) na Hipertensão Pulmonar: Dados da Prática Clínica Real em 3 Anos de Acompanhamento

**DOI:** 10.36660/abc.20210492

**Published:** 2022-05-04

**Authors:** Fernanda Brum Spilimbergo, Taís Silveira Assmann, Marcelo Bellon, Laís Machado Hoscheidt, Cássia Ferreira Braz Caurio, Márcia Puchalski, Bruno Hochhegger, Gabriela Roncato, Gisela Martina Bohns Meyer

**Affiliations:** 1 Centro de Hipertensão Pulmonar Complexo Hospitalar Santa Casa de Misericórdia de Porto Alegre Porto Alegre RS Brasil Centro de Hipertensão Pulmonar, Complexo Hospitalar Santa Casa de Misericórdia de Porto Alegre, Porto Alegre, RS – Brasil; 2 Bayer S.A. São Paulo SP Brasil Bayer S.A., São Paulo, SP – Brasil

**Keywords:** Hipertensão Arterial Pulmonar, Hipertensão Pulmonar, Pressão Propulsora Pulmonar

## Abstract

**Fundamento:**

A hipertensão pulmonar (HP) é uma doença rara e complexa com prognóstico ruim, que exige tratamento pela vida toda.

**Objetivo:**

Descrever dados de 3 anos de acompanhamento da vida real sobre o tratamento com estimuladores de guanilato ciclase solúvel (Riociguate) de pacientes com HP, medindo parâmetros atuais de avaliação de risco.

**Métodos:**

Coletamos dados clínicos e epidemiológicos retrospectivamente de pacientes com HP do grupo 1 (hipertensão arterial pulmonar) e do grupo 4 (HP tromboembólica crônica). Parâmetros não invasivos e invasivos correspondentes à avaliação de risco foram analisados na linha de base e no acompanhamento. Foram realizadas análises estatísticas usando o software SPSS 18.0, e os p-valores <0,050 foram considerados estatisticamente significativos.

**Resultados:**

No total, 41 pacientes tratados com riociguate foram incluídos no estudo. Entre eles, 31 já concluíram 3 anos de tratamento e foram selecionados para a seguinte análise. Na linha de base, 70,7% dos pacientes estavam nas classes funcionais III ou IV da OMS. Depois de 3 anos de tratamento, a classe funcional da OMS melhorou significativamente em todos os pacientes. Além disso, a mediana do teste de caminhada de 6 minutos (TC6M) aumentou significativamente de 394 ± 91 m na linha de base para 458 ± 100 m após 3 anos de acompanhamento (p= 0,014). O índice de sobrevida após três anos foi de 96,7%.

**Conclusão:**

Em nossa coorte de vida real, a maioria dos pacientes com HP tratados com riociguate demonstraram parâmetros de risco estáveis ou melhores, especialmente no TC6M, aos 3 anos de acompanhamento.

## Introdução

A hipertensão pulmonar (HP) é uma condição clínica progressiva caracterizada pela elevação da pressão arterial pulmonar média (PAPm) acima de 20 mmHg em repouso^1^ Antes da era moderna da terapia para HP, a expectativa de vida média após o diagnóstico era 2,8 anos para adultos com HP.^2^ O desenvolvimento e a disponibilidade de novas terapias aumentaram significativamente a qualidade de vida e a sobrevida de pacientes com HP.^3 , 4^

A HP é classificada em cinco subgrupos clínicos: hipertensão arterial pulmonar (HAP), HP devido a doença cardíaca esquerda, HP devido a doença pulmonar crônica; HP tromboembólica crônica (HPTEC), e HP com mecanismos multifatoriais e/ou pouco claros.^3^ Essa categorização considera a apresentação clínica similar, achados patológicos, características hemodinâmicas, e estratégia de tratamento.^5^ Especificamente, a HAP (grupo 1) e HPTEC (grupo 4) são caracterizadas como HP pré-capilares, com pressão de oclusão da artéria pulmonar ≤15 mmHg e resistência vascular pulmonar (RVP) ≥3 unidades Wood.1 Embora a HPTEC tenha origem um tromboembolismo pulmonar crônico, as doenças HAP e HPTEC apresentam perda e remodelagem obstrutiva do leito vascular pulmonar, resultando em pressão arterial pulmonar elevada e RVP, insuficiência cardíaca direita progressiva e morte.^6^

Além de apresentar semelhanças fisiopatológicas, a HAP e a HPTEC também têm semelhanças no tratamento farmacológico. A endarterectomia pulmonar ainda é o tratamento de escolha para pacientes com HPTEC cirúrgica; entretanto, para aqueles considerados inoperáveis, a evidência científica justificar o início de uma terapia médica e a consideração de angioplastia pulmonar por balão.^7^

O estimulador de guanilato ciclase solúvel (riociguate) tem um modo de ação duplo: 1) estimula diretamente a guanilato ciclase solúvel independentemente do óxido nítrico e 2) aumenta a sensibilidade da guanilato ciclase solúvel ao óxido nítrico.^8 , 9^ Como se sabe que pacientes com HAP ou HPTEC têm níveis reduzidos de óxido nítrico,^10^ esse modo de ação é muito importante para melhorar a dinâmica da vasculatura pulmonar. Estudos anteriores demonstraram que o riociguate melhorou significativamente a capacidade de exercício, bem como desfechos secundários, tais como RVP, classe funcional da Organização Mundial de Saúde (OMS) e peptídeo natriurético pró-cerebral N-terminal (NT-proBNP) em pacientes com HAP^11^ e HPTEC.^12^ Com base nesses resultados, o riociguate foi aprovado para tratamento de adultos com HAP em monoterapia ou em combinação,^5^ e é o único medicamento aprovado por agências regulatórias americanas, europeias e brasileiras para o tratamento de HPTEC inoperável ou HP residual.^13 , 14^ Nesse contexto, o objetivo desse estudo foi descrever dados de vida real do tratamento de pacientes com HP do grupo 1 (HAP) e do grupo 4 (HPTEC) com riociguate no Brasil, medindo parâmetros de avaliação de risco atuais.

## Métodos

### Seleção dos pacientes

**Figura 1:**
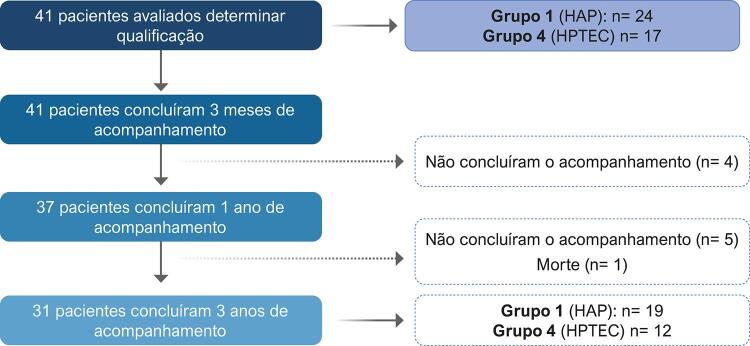
– Fluxograma dos pacientes durante o estudo. HAP: hipertensão arterial pulmonar; HPTEC: hipertensão pulmonar tromboembólica crônica.

Todos os pacientes com HAP e HPTEC que iniciaram o tratamento com riociguate entre 2010 e 2020 no Centro de Hipertensão Pulmonar, Complexo Hospitalar Santa Casa de Porto Alegre foram incluídos e analisados retrospectivamente ( Figura 1 ). Trata-se de um centro de referência para o tratamento da HP que participa dos principais estudos clínicos multicêntricos na região desde 2005. Este estudo foi aprovado pelo comitê de ética local (número: 30199714.6.0000.5335). O diagnóstico da HP foi confirmado por um cateterismo do coração direito (CCD) em todos os pacientes.

### Procedimentos

Características demográficas e clínicas foram coletadas na linha de base, aos 3 meses, a 1 ano, e aos 3 anos de acompanhamento. Esses parâmetros incluíram a determinação da etiologia da HP, classe funcional da OMS, teste da caminhada de seis minutos (TC6M), NT-proBNP, e medições hemodinâmicas.

A linha de base foi definida no momento da estabilidade do medicamento antes de iniciar o tratamento com riociguate. A classe funcional da OMS foi determinada pelo médico atendente em cada visita. O TC6M foi realizado de acordo com as diretrizes da ATS.^15^ O CCD foi realizado usando-se um cateter Swan-Ganz. O débito cardíaco foi medido por termodiluição. A sobrevida foi estabelecida com base em registros médicos eletrônicos.

### Análise estatística

A distribuição normal foi verificada pelo teste de Shapiro-Wilk. As variáveis contínuas com distribuição normal são expressas como médias ± desvio padrão (DP). Variáveis com distribuição distorcida passaram por transformação logarítmica antes das análises e são apresentadas como medianas (25º – 75º percentis).^16^ Dados categóricos são expressos como números absolutos e porcentagens.

Características clínicas, laboratoriais e hemodinâmicas foram comparadas entre os grupos (HAP e HPTEC) por testes t de Student não pareado^16^ ou χ^2^, conforme apropriado. As diferenças entre linha de base, aos 3 meses, a 1 ano, e aos 3 anos de acompanhamento foram comparadas pelo teste t de Student pareado. Todas as análises estatísticas foram realizadas utilizando-se testes de correlação de Pearson. Foram realizadas análises estatísticas usando o software SPSS 18.0 (SPSS, Chicago, IL), e os p-valores <0,050 foram considerados estatisticamente significativos.

## Resultados

Um total de 41 pacientes que haviam sido tratados com riociguate se qualificaram para a análise. Entre eles, 31 já tinham concluído 3 anos de tratamento e foram selecionados para a seguinte análise ( Figura 1 ).

As características demográficas e clínicas de linha de base da população do estudo são apresentadas na Tabela 1 . Dos 41 pacientes cadastrados neste estudo, 24 pacientes foram classificados como portadores de HAP (grupo 1) e 17 pacientes, como portadores de HPTEC (grupo 4). As etiologias mais comuns da HAP foram idiopáticas (67%). Os pacientes eram predominantemente do sexo feminino (70,7%), com uma idade média no momento do diagnóstico de HP de 42,2 ± 3,5 anos. A maioria dos participantes apresentaram manifestações moderadas a graves da doença na linha de base, com 70,7% dos pacientes apresentando classe funcional da OMS III ou IV. No geral, os níveis medianos de NT-proBNP foram 655 pg/ml e a média do TC6M foi de 386 metros. Hemodinamicamente, os pacientes apresentaram uma PAPm de 45,5 ± 11,7 mmHg; RVP de 9,8 ± 1,0 Wood; índice cardíaco (IC) de 2,7 ± 0,1 L/min ( Tabela 1 ). É importante notar que não há diferença entre os grupos de HAP e HPTEC em relação às características analisadas ( Tabela 1 ).

**Tabela 1:** – Características de linha de base de pacientes tratados com riociguate

Características da linha de base	Total (n= 41)	HAP (n= 24)	HPTEC (n= 17)	P- *valor* *
**Sexo, n (% masculino)**	12 (29,3)	7 (29,1)	5 (29,4)	0,889
**Idade no diagnóstico, anos**	42,2 ± 3,5	40,0 ± 4,3	55,7 ± 15,1	0,514
**IMC (kg/m^2^)**	27,3 ± 1,5	26,7 ± 4,6	29,0 ± 1,5	0,732
**Classificação HAP (n)**				
Idiopática	-	16	-	-
Familiar	-	1	-	
Associada a doença do tecido conjuntivo	-	4	-	
Associada a doença cardíaca congênita	-	1	-	
Associada a uso de anorexígeno ou anfetamina	-	1	-	
Associada a HIV	-	1	-	
**Classe funcional OMS, n (%)**				
II	12 (29,3)	7 (29,2)	5 (29,5)	0,087
III	26 (63,4)	17 (70,8)	9 (52,9)	
IV	3 (7,3)	0 (0,0)	3 (17,6)	
**Medicamentos para HP concomitantes, n (%)**				
Antagonista do receptor da endotelina	18	14 (77,8)	4 (22,2)	0,080
Prostanoide	2	1 (50,0)	1 (50,0)	0,999
Anticoagulante	17	10 (58,8)	7 (41,2)	0,999
Diuréticos	15	9 (60,0)	6 (30,0)	0,999
**Distância na caminhada de 6 minutos (m)**	386,1 ± 99,2	410,4 ± 72,4	346,5 ± 136,5	0,201
**NT-proBNP (pg/mL)**	655 (127 - 1191)	190 (90 – 1028)	793 (259 - 2554)	0,570
**PAP sistólica (mmHg)**	81,1 ± 3,0	79,9 ± 18,3	82,9 ± 21,3	0,487
**PAP diastólica (mmHg)**	36,2 ± 1,7	38,8 ± 11,7	33,8 ± 6,6	0,121
**PAPm (mmHg)**	45,5 ± 11,7	55,4 ± 13,4	44,6 ± 8,4	0,410
**POAP (mmHg)**	7,8 ± 0,4	7,3 ± 0,5	9,5 ± 0,3	0,131
**RVP**	9,8 ± 1,0	11,4 ± 0,8	9,0 ± 0,5	0,211
**Índice cardíaco (L/min)**	2,7 ± 0,1	2,7 ± 0,8	2,5 ± 0,8	0,921
**Débito cardíaco (L/min)**	4,9 ± 0,3	4,7 ± 1,3	4,9 ± 0,7	0,778

Os resultados são apresentados como média ± DP, n (%), ou mediana (25º - 75º), conforme apropriado. HPTEC: hipertensão pulmonar tromboembólica crônica; PAPm: pressão arterial pulmonar média; NT-proBNP: Peptídeo natriurético pró-cerebral N-terminal; IMC: índice de massa corporal; HAP: hipertensão arterial pulmonar; PAP: pressão arterial pulmonar; POAP: pressão de oclusão da artéria pulmonar; RVP: resistência vascular pulmonar; OMS: Organização Mundial de Saúde. *p-valor calculado usando o teste χ^2^ ou o Teste t de Student não pareado para comparar com as características da linha de base entre os grupos de HAP e HPTEC, conforme apropriado.

**Figura 2:**
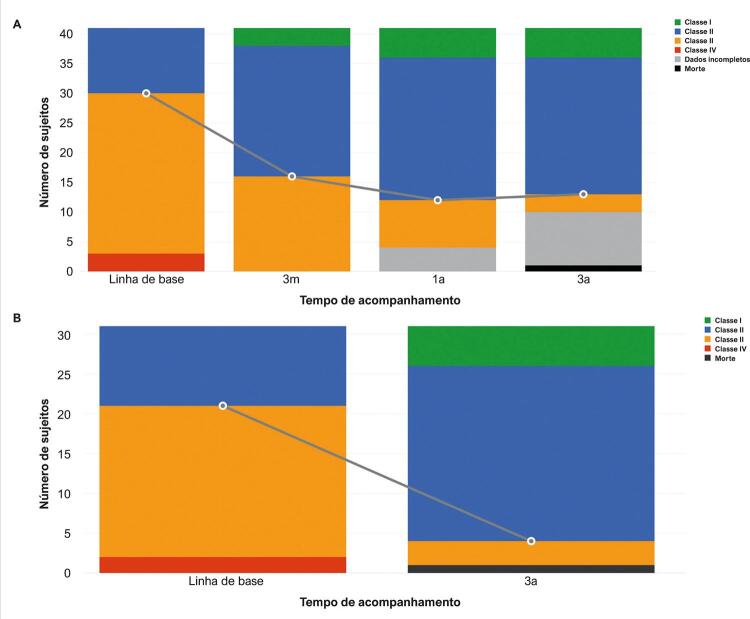
– Alteração ao longo do tempo na classe funcional da OMS para pacientes com hipertensão pulmonar. A) Dados de todos os 41 pacientes na linha de base e nos períodos de acompanhamento. B) Dados de todos os 31 pacientes que concluíram 3 anos de acompanhamento.

Durante os 3 anos de acompanhamento do paciente, observou-se uma melhoria da capacidade funcional, conforme ilustrado na Figura 2 . Durante o acompanhamento, o número de pacientes na classe funcional III diminuiu, e o da classe funcional II aumentou ( Figura 2a ). Considerando apenas os pacientes que concluíram 3 anos de acompanhamento (n= 31), na linha de base, 61% dos pacientes estavam na classe funcional III e após 3 anos de tratamento com riociguate, 10% dos pacientes continuaram na classe funcional III. Da mesma forma, na linha de base, 32% dos pacientes estavam na classe funcional II e, depois do tratamento, 71% dos pacientes estavam na classe funcional II. Particularmente, o número de pacientes na classe funcional I aumentou de 0, na linha de base, para 5, após 3 anos de tratamento ( Figura 2b ).

**Figura 3:**
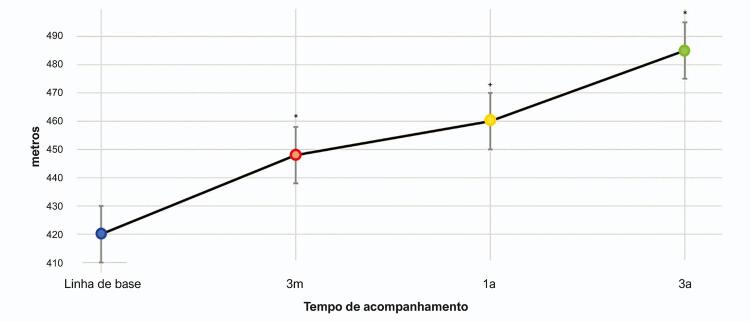
– Alteração ao longo do tempo no teste da caminhada de seis minutos (TC6M) em pacientes com hipertensão pulmonar. *p-valor< 0,05; +p-valor< 0,10; Teste t de Student pareado comparado com a linha de base.

**Figura 4:**
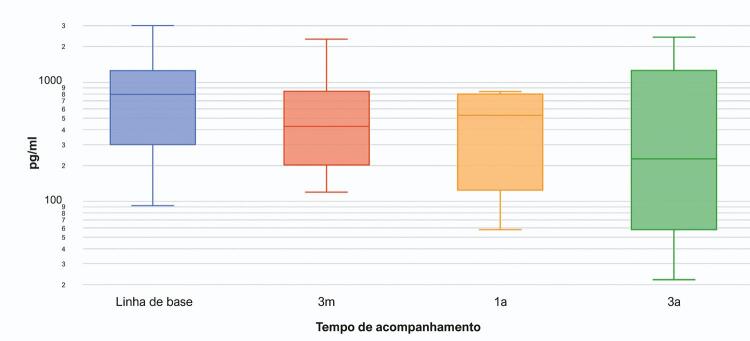
– Alteração ao longo do tempo no fragmento do Peptídeo natriurético pró-cerebral N-terminal (NT-proBNP) para pacientes com hipertensão pulmonar.

As características clínicas dos 31 pacientes que concluíram 3 anos de acompanhamento estão descritas na Tabela 2 . Nossos resultados demonstraram uma melhora significativa de 64 m após 3 anos de tratamento com riociguate em comparação com a linha de base (p= 0,014). Após a estratificação por etiologia de HP, observou-se uma redução de 59 m nos pacientes com HAP (p= 0,045) e de 70 m em pacientes com HPTEC (p= 0,080). Além disso, conforme mostrado na Figura 3 , o TC6M melhorou significativamente a 3 meses, a 1 ano, e aos 3 anos, em comparação com os resultados da linha de base. Embora a redução nos níveis de NT-proBNP não seja estatisticamente significativa, pode-se observar uma redução clinicamente importante de 663 pg/ml nos níveis de NT-proBNP após o tratamento com riociguate ( Tabela 2 e Figura 4 ). Ademais, há uma correlação negativa entre o TC6M e os níveis de NT-proBNP após 3 anos de acompanhamento (r= -0,520, p= 0,027). Não se observou nenhuma alteração significativa em PAD ou IC nas aferições de linha de base em comparação com o acompanhamento de 3 anos. De acordo com a estratificação de risco não invasiva francesa, nenhum paciente tinha risco baixo na linha de base e 7 pacientes chegaram ao status de risco baixo após 3 anos de tratamento. Durante o período de acompanhamento, um único paciente (3,2%) morreu devido a causas associadas a HP, e essa morte ocorreu em um paciente com classe funcional III na linha de base.

**Tabela 2:** – Alterações em aferições clínicas e laboratoriais após 3 anos de tratamento com riociguate

Característica	Linha de base (n= 31)	3 anos (n= 31)	Δ	p-valor*
**PAP sistólica (mmHg)**	81,6 ± 16,1	78,2 ± 14,2	-3,4	0,500
**PAP diastólica (mmHg)**	35,1 ± 5,2	34,2 ± 4,7	-0,9	0,618
**PAPm (mmHg)**	43,5 ± 9,0	39,6 ± 3,4	-3,9	0,253
**POAP (mmHg)**	7,3 ± 1,8	9,6 ± 3,1	2,3	0,013
**RVP**	9,3 ± 3,0	7,9 ± 3,1	-1,4	0,157
**Índice cardíaco (L/min)**	2,9 ± 0,8	2,7 ± 0,7	-0,2	0,170
**Débito cardíaco (L/min)**	5,2 ± 1,5	5,0 ± 1,5	-0,2	0,504
**Distância na caminhada de 6 minutos (m)**	394 ± 91	458 ± 100	64	0,014
**NT-proBNP (pg/mL)**	793 (145 - 1235)	130 (58 - 980)	-663	0,197

Os resultados são apresentados como média ± DP, ou mediana (25º - 75º), conforme apropriado. PAPm: pressão arterial pulmonar média; NT-proBNP: Peptídeo natriurético pró-cerebral N-terminal; PAP: pressão arterial pulmonar; POAP: pressão de oclusão da artéria pulmonar; RVP: resistência vascular pulmonar. *p-valor calculado usando o teste t de Student pareado em comparação à linha de base.

Além disso, nosso centro também observou os resultados de um subgrupo de 10 pacientes que completaram 10 anos de uso de riociguate. Na mesma linha dos resultados de 3 anos de acompanhamento, os status clínicos desses pacientes também foram satisfatórios com baixo risco e boa tolerância ao tratamento.

## Discussão

Até onde sabemos, este é o primeiro estudo a detalhar a experiência em vida real de tratamento de HAP e HPTEC com riociguate por pelo menos 3 anos. Nessa coorte da vida real, demonstrou-se uma melhoria no TC6M e na classe funcional da OMS nos dois grupos, HAP e HPTEC.

O TC6M é uma ferramenta simples para a avaliação da capacidade funcional de exercício, que reflete a capacidade do indivíduo de desempenhar atividades de rotina. Além disso, os pacientes estão familiarizados com ela^5^ e ela foi o desfecho mais utilizado principalmente em ensaios clínicos de terapias de HP.^17^ Entre os testes ergométricos, o TC6M demonstrou ter a melhor capacidade de capturar as alterações na capacidade de exercício além de demonstrar ser um preditor independente de morbidade e mortalidade na HP.^18 - 20^

Nossos resultados demonstraram uma melhora significativa de 64 m após 3 anos de tratamento com riociguate, que está de acordo com os achados de melhorias em TC6M em vários estudos, em ensaios randomizados controlados^11 , 12^ bem como em estudos de extensão,^21 , 22^ de rótulo aberto^23 , 24^ e de vida real.^25^ Além disso, nossos dados apresentaram um aumento gradual da distância de TC6M, de 3 meses a 3 anos após o início do tratamento, com uma mediana final acima de 440 m, que é considerado um status de baixo risco para os pacientes.^5^

As diretrizes de tratamento de 2015 da Sociedade Europeia de Cardiologia ( *European Society of Cardiology* –ESC)/Sociedade Europeia Respiratória (European Respiratory Society – ERS) recomendam avaliações de risco regulares em pacientes de HAP, para controlar os pacientes com foco no baixo risco.^5^ A avaliação de risco é realizada usando-se uma abordagem multidimensional, mas há versões abreviadas, tais como o método não invasivo de registro francês, que avalia TC6M, NT-proBNP e classe funcional da OMS.^17^ Nesse contexto, também foram identificadas melhorias em NT-proBNP e na classe funcional da OMS em nossos pacientes tratados com riociguate. Além disso, sete pacientes alcançaram o status de baixo risco. Esses resultados enfatizam os benefícios do medicamento para alcançar os objetivos do tratamento e, talvez, reduzir a mortalidade de 1 ano estimada. Relatórios anteriores encontraram melhorias significativas nesses parâmetros^11 , 12^ e o alcance do escore de baixo risco^17^ após o tratamento com riociguate. Nossos dados provavelmente não alcançaram a significância estatística devido ao tamanho pequeno da amostra.

Nosso estudo teve algumas limitações. Primeiramente, devido ao desenho de coorte de vida real de nosso estudo, o número de pacientes em cada visita variou. Segundo, esse é um estudo retrospectivo com uma amostra reduzida. Terceiro, os resultados vêm de um centro único. Portanto, essas limitações devem ser consideradas ao se interpretar os resultados.

## Conclusão

Em nossa coorte de vida real, a maioria dos pacientes com HP tratados com riociguate demonstraram parâmetros de risco estáveis ou melhores, especialmente o TC6M, aos 3 anos de acompanhamento. Além disso, nossos dados conseguiram reproduzir os resultados de estudos fundamentais durante nosso acompanhamento.
