# Supplementary material for: Estimuladores de Guanilato Ciclase Solúvel (Riociguate) na Hipertensão Pulmonar: Dados da Prática Clínica Real em 3 Anos de Acompanhamento
Source: Arq Bras Cardiol. 2022 May 4;118(6):1059–66. [Article in Portuguese] doi: 10.36660/abc.20210492 (PMC9345144; doi:10.36660/abc.20210492)
Supplement: Translation

# Translation: Soluble Guanylate Cyclase Stimulators (Riociguat) in Pulmonary Hypertension: Data from Real-Life Clinical Practice in a 3-Year Follow-Up

**Authors:** Fernanda Brum Spilimbergo, Taís Silveira Assmann, Marcelo Bellon, Laís Machado Hoscheidt, Cássia Ferreira Braz Caurio, Márcia Puchalski, Bruno Hochhegger, Gabriela Roncato, Gisela Martina Bohns Meyer

**Affiliations:** 1 Brazil Centro de Hipertensão Pulmonar, Complexo Hospitalar Santa Casa de Misericórdia de Porto Alegre, Porto Alegre, RS – Brazil; 2 Brazil Bayer S.A., São Paulo, SP – Brazil

**Keywords:** Pulmonary Arterial Hypertension, Hypertension, Pulmonary, Pulmonary Wedge Pressure

## Abstract

**Background:**

Pulmonary hypertension (PH) is a rare and complex disease with poor prognosis, which requires lifelong treatment.

**Objective:**

To describe 3-year follow-up real-life data on treatment with soluble guanylate cyclase stimulators (Riociguat) of patients with PH, measuring current risk assessment parameters.

**Methods:**

This study retrospectively collected clinical and epidemiological data of patients with PH of group 1 (pulmonary arterial hypertension) and group 4 (chronic thromboembolic PH). Non-invasive and invasive parameters corresponding to the risk assessment were analyzed at baseline and follow-up. Statistical analyses were performed using the SPSS 18.0 software, and p-values < 0.050 were considered statistically significant.

**Results:**

In total, 41 patients receiving riociguat were included in the study. Of them, 31 had already completed 3 years of treatment and were selected for the following analysis. At baseline, 70.7% of patients were in WHO functional class III or IV. After 3 years of treatment, the WHO functional class significantly improved in all patients. In addition, the median of the 6-minute walk test (6MWT) significantly increased from 394 ± 91 m at baseline to 458 ± 100 m after 3 years of follow-up (p= 0.014). The three-year survival rate was 96.7%.

**Conclusion:**

In our real-life cohort, most patients with PH treated with riociguat showed stable or improved risk parameters, especially in the 6MWT, at 3 years of follow-up.

## Introduction

Pulmonary hypertension (PH) is a progressive clinical condition, characterized by the elevation of mean pulmonary arterial pressure (mPAP) to greater than 20 mmHg when at rest.^1^ Prior to the modern era of PH therapy, the average life expectancy after diagnosis had been 2.8 years for adults with PH.2 The development and availability of new therapies have significantly improved the quality of life and the survival of PH patients.^3 , 4^

PH is classified into five clinical subgroups: pulmonary arterial hypertension (PAH), PH due to left-sided heart disease, PH due to chronic lung disease, chronic thromboembolic PH (CTEPH), and PH with an unclear and/or multifactorial mechanisms.^3^ This categorization considers a similar clinical presentation, pathological findings, hemodynamic characteristics, and treatment strategy.^5^ Specifically, PAH (group 1) and CTEPH (group 4) are characterized as pre-capillary PH, with pulmonary arterial wedge pressure ≤15 mmHg and pulmonary vascular resistance (PVR) ≥3 Wood Units.^1^ Although CTEPH originates from a chronic pulmonary thromboembolism, PAH and CTEPH diseases present loss and obstructive remodeling of the pulmonary vascular bed, resulting in elevated pulmonary arterial pressure and PVR, progressive right heart failure, and death.^6^

In addition to presenting pathophysiological similarities, PAH and CTEPH also have similarities in pharmacological treatment. Pulmonary endarterectomy remains the treatment of choice for patients with surgical CTEPH; however, for those considered inoperable, scientific evidence supports the initiation of medical therapy and the consideration of balloon pulmonary angioplasty.^7^

The soluble guanylate cyclase stimulator (riociguat) has a dual mode of action: 1) it directly stimulates soluble guanylate cyclase independently of nitric oxide and 2) it increases the sensitivity of soluble guanylate cyclase to nitric oxide.^8 , 9^ As it is known that patients with PAH or CTEPH have reduced levels of nitric oxide,^10^ this mode of action is very important to improve the dynamics of the pulmonary vasculature. Previous studies have shown that riociguat significantly improved exercise capacity, as well as secondary endpoints, such as PVR, the World Health Organization (WHO) functional class, and N-terminal pro-brain natriuretic peptide (NT-proBNP) in patients with PAH^11^ and CTEPH.^12^ Based on these results, riociguat was approved for the treatment of adults with PAH in monotherapy or in combination,^5^ and it is the only medication approved by American, European, and Brazilian regulatory agencies for the treatment of inoperable CTEPH or residual PH.^13 , 14^ In this context, the aim of this study was to describe real-life data on the treatment of patients with PH from group 1 (PAH) and group 4 (CTEPH) with riociguat in Brazil, measuring current risk assessment parameters.

## Methods

### Selection of patients

All patients with PAH and CTEPH who started the treatment with riociguat between 2010 and 2020 at the Centro de Hipertensão Pulmonar, Complexo Hospitalar Santa Casa de Porto Alegre were included and analyzed retrospectively ( Figure 1 ). This is a Reference Center for PH treatment, which participates in the main multicenter clinical studies in the area since 2005. This study was approved by the local ethics committee (number: 30199714.6.0000.5335). Diagnosis of PH was confirmed by right heart catheterization (RHC) in all patients.

**Figure 1:** – Flowchart of patients throughout the study. PAH: pulmonary arterial hypertension; CTEPH: chronic thromboembolic pulmonary hypertension.

### Procedures

Demographic and clinical characteristics were collected at baseline, 3 months, 1 year, and 3 years of follow-up. These parameters included the determination of PH etiology, the WHO functional class, the 6-minute walking test (6MWT), NT-proBNP, and hemodynamic measurements.

Baseline was defined as the time of stable medication before starting treatment with riociguat. The WHO functional class was determined by the treating physician at each visit. The 6MWT was carried out according to ATS guidelines.^15^ RHC was performed using a Swan-Ganz catheter. Cardiac output was measured by thermodilution. Survival was established based on the electronic medical records.

### Statistical analysis

Normal distribution was checked using the Shapiro-Wilk test. The continuous variables with normal distribution are presented as mean ± standard deviation (SD). Variables with skewed distribution were log-transformed before analyses and are presented as medians (25th – 75th percentiles).^16^ Categorical data are shown as absolute number and percentages.

Clinical, laboratorial, and hemodynamics characteristics were compared between groups (PAH and CTEPH), using the unpaired Student’s *t* -test^16^ or χ^2^tests, as appropriate. Differences between baseline, 3 months, 1 year, and 3 years of follow-up were compared using the paired Student’s *t* -test. Correlation analyses were performed using Pearson’s correlation tests. Statistical analyses were performed using the SPSS 18.0 software (SPSS, Chicago, IL), and p-values < 0.050 were considered statistically significant.

## Results

A total of 41 patients who had been treated with riociguat were eligible for the analysis. Of these, 31 had completed 3 years of follow-up and were selected for the following analysis ( Figure 1 ).

Baseline demographic and clinical characteristics of the study population are shown in Table 1 . Of the 41 patients enrolled in this study, 24 patients were classified as PAH (group 1) and 17 patients as CTEPH (group 4). The most common PAH etiologies were idiopathic (67%). Patients were predominantly female (70.7%), with a mean age at PH diagnosis of 42.2 ± 3.5 years. Most participants showed moderate to severe disease manifestations at baseline, with 70.7% of the patients presenting WHO functional class III or IV. Overall, the median levels of NT-proBNP were 655 pg/ml, and the mean 6MWT was 386 meters. Hemodynamically, patients showed mPAP of 45.5 ± 11.7 mmHg; PVR of 9.8 ± 1.0 Wood; and a cardiac index (CI) of 2.7 ± 0.1 L/min ( Table 1 ). It is important to note that no difference was found between the PAH and CTEPH groups regarding the analyzed characteristics ( Table 1 ).

**Table 1:** – Baseline characteristics of patients treated with riociguat

Baseline characteristics	Total (n= 41)	PAH (n= 24)	CTEPH (n= 17)	p-value*
**Gender, n (% male)**	12 (29.3)	7 (29.1)	5 (29.4)	0.889
**Age at diagnosis, years**	42.2 ± 3.5	40.0 ± 4.3	55.7 ± 15.1	0.514
**BMI (kg/m^2^)**	27.3 ± 1.5	26.7 ± 4.6	29.0 ± 1.5	0.732
**PAH classification (n)**				
Idiopathic	-	16	-	-
Familiar	-	1	-	
Associated with connective-tissue disease	-	4	-	
Associated with congenital heart disease	-	1	-	
Associated with anorexigen or amphetamine use	-	1	-	
Associated with HIV	-	1	-	
**WHO functional class, n (%)**				
II	12 (29.3)	7 (29.2)	5 (29.5)	0.087
III	26 (63.4)	17 (70.8)	9 (52.9)	
IV	3 (7.3)	0 (0.0)	3 (17.6)	
**Concomitant PH medications, n (%)**				
Endothelin-receptor antagonist	18	14 (77.8)	4 (22.2)	0.080
Prostanoid	2	1 (50.0)	1 (50.0)	0.999
Anticoagulant	17	10 (58.8)	7 (41.2)	0.999
Diuretics	15	9 (60.0)	6 (30.0)	0.999
**6-min walking distance (m)**	386.1 ± 99.2	410.4 ± 72.4	346.5 ± 136.5	0.201
**NT-proBNP (pg/ml)**	655 (127 - 1191)	190 (90 – 1028)	793 (259 - 2554)	0.570
**Systolic PAP (mmHg)**	81.1 ± 3.0	79.9 ± 18.3	82.9 ± 21.3	0.487
**Diastolic PAP (mmHg)**	36.2 ± 1.7	38.8 ± 11.7	33.8 ± 6.6	0.121
**mPAP (mmHg)**	45.5 ± 11.7	55.4 ± 13.4	44.6 ± 8.4	0.410
**PAWP (mmHg)**	7.8 ± 0.4	7.3 ± 0.5	9.5 ± 0.3	0.131
**PVR**	9.8 ± 1.0	11.4 ± 0.8	9.0 ± 0.5	0.211
**Cardiac index (L/min)**	2.7 ± 0.1	2.7 ± 0.8	2.5 ± 0.8	0.921
**Cardiac output (L/min)**	4.9 ± 0.3	4.7 ± 1.3	4.9 ± 0.7	0.778

Results are presented as mean ± SD, n (%), or median (25^th^- 75^th^), as appropriate. CTEPH: chronic thromboembolic pulmonary hypertension; mPAP: mean pulmonary artery pressure; NT-proBNP: N-terminal pro-brain natriuretic peptide; BMI: body mass index; PAH: pulmonary arterial hypertension; PAP: pulmonary artery pressure; PAWP: pulmonary arterial wedge pressure; PVR: pulmonary vascular resistance; WHO: World Health Organization. *p-value computed using χ^2^test or unpaired Student’s t-test to compare the baseline characteristics between PAH and CTEPH groups, as appropriate.

During a 3-year patient follow-up, a functional capacity improvement was noted, as illustrated in Figure 2 . During the follow-up, the number of patients in functional class III decreased, and that of functional class II increased ( Figure 2a ). Considering only the patients who completed 3 years of follow-up (n= 31), at baseline, 61% patients were functional class III, and after 3 years of treatment with riociguat, 10% of patients continued as functional class III. In the same way, at baseline, 32% of the patients were functional class II, and after treatment, 71% of the patients were in functional class II. In particular, the number of patients in functional class I increased from 0 at baseline to 5 patients after 3 years of treatment ( Figure 2b ).

**Figure 2:** – Change of WHO functional class over time in patients with pulmonary hypertension. A) Data from all 41 patients in baseline and follow-up periods. B) Data from the 31 patients who completed 3 years of follow-up.

Clinical characteristics of the 31 patients who completed the 3 years of follow-up are described in Table 2 . Our results showed a significant improvement of 64 m after 3 years of treatment with riociguat when compared to the baseline (p= 0.014). After stratification by PH etiology, a reduction of 59 m was observed in PAH (p= 0.045) and of 70 m in CTEPH patients (p= 0.080). Moreover, as shown in Figure 3 , 6MWT significantly improved in 3 months, 1 year, and 3 years when compared to baseline results. Although the decrease in NT-proBNP levels is not statically significant, a clinically important reduction of 663 pg/ml could be observed in NT-proBNP levels after treatment with riociguat ( Table 2 and Figure 4 ). Moreover, there is a negative correlation between 6MWT and NT-proBNP levels after 3 years of follow-up (r= -0.520, p= 0.027). No significant changes were observed in RAP and CI in baseline measurements when compared to 3 years of follow-up. According to the French non-invasive risk stratification, no patient was at low risk at baseline and 7 patients achieved low risk status after 3 years of treatment. During the follow-up period, a total of one (3.2%) patient died of PH-related causes; and this death occurred in patient with functional class III at baseline.

**Table 2:** – Changes in clinical and laboratorial measurements after 3 years of treatment with riociguat

Characteristic	Baseline (n= 31)	3 years (n= 31)	Δ	p-value*
**Systolic PAP (mmHg)**	81.6 ± 16.1	78.2 ± 14.2	-3.4	0.500
**Diastolic PAP (mmHg)**	35.1 ± 5.2	34.2 ± 4.7	-0.9	0.618
**mPAP (mmHg)**	43.5 ± 9.0	39.6 ± 3.4	-3.9	0.253
**PAWP (mmHg)**	7.3 ± 1.8	9.6 ± 3.1	2.3	0.013
**PVR**	9.3 ± 3.0	7.9 ± 3.1	-1.4	0.157
**Cardiac Index (L/min)**	2.9 ± 0.8	2.7 ± 0.7	-0.2	0.170
**Cardiac output (L/min)**	5.2 ± 1.5	5.0 ± 1.5	-0.2	0.504
**6-min walk distance (m)**	394 ± 91	458 ± 100	64	0.014
**NT-proBNP (pg/ml)**	793 (145 - 1235)	130 (58 - 980)	-663	0.197

Results are presented as mean ± SD or median (25^th^- 75^th^), as appropriate. mPAP: mean pulmonary artery pressure; NT-proBNP: N-terminal pro-brain natriuretic peptide; PAP: pulmonary artery pressure; PAWP: pulmonary arterial wedge pressure; PVR: pulmonary vascular resistance. *p-value computed using paired Student’s t-test compared to Baseline.

**Figure 3:** – Change of 6-min walking test (6MWT) over time in patients with pulmonary hypertension. *p-value< 0.05; +p-value< 0.10; Paired Student’s t-test compared to Baseline.

**Figure 4:** – Change of N-terminal pro-brain natriuretic peptide (NT-proBNP) over time in patients with pulmonary hypertension.

Additionally, our center also observed the results of a sub-group of 10 patients who have completed 10-years using riociguat. In the same line of 3 years of follow-up results, the clinical status of these patients was also satisfactory, with low risk and good treatment tolerability.

## Discussion

To the best of our knowledge, this is the first study to detail the real-life experience of treating PAH and CTEPH with riociguat for at least 3 years. In this real-life cohort, we show an improvement in 6MWT and WHO functional class in both groups, PAH and CTEPH.

The 6MWT is a simple tool for the evaluation of functional exercise capacity, which reflects the capacity of the individual to perform activities of daily living. Moreover, it is familiar to patients^5^ and has been the most employed primary endpoint in clinical trials of PH therapies.^17^ Among exercise tests, the 6MWT has proven to have the best ability to capture changes in exercise capacity and has regularly proven to be an independent predictor of morbidity and mortality in PH.^18 - 20^

Our results showed a significant improvement of 64 m after 3 years of treatment with riociguat, which is in accordance with the findings of improvements in 6MWT of many studies, both randomized controlled trials^11 , 12^ as well as extension,^21 , 22^ open-label^23 , 24^ and real-life studies.^25^ In addition, our data presented a gradual increase in 6MWT distance, from 3 months to 3 years after the start of treatment, with a final median greater than 440m, which is considered a low-risk status for patients.^5^

The 2015 European Society of Cardiology (ESC)/European Respiratory Society (ERS) treatment guidelines recommend regular risk assessment in PAH patients, to manage the patients, focusing on low risk.^5^ This risk assessment is performed using a multidimensional approach, but there are abbreviated versions, such as the French registry non-invasive method, which evaluate 6MWT, NT-ProBNP, and WHO functional class.^17^ In this context, we also found improvements in NT-ProBNP and WHO functional class in our patients treated with riociguat. Moreover, seven patients achieved the low-risk status. These results emphasize the benefits of medication to the achievement of treatment goals and, perhaps, to reduce the estimated 1-year mortality. Previous reports found significant improvements in these parameters^11 , 12^ and in the low-risk score achievement^17^ after treatment with riociguat. It is likely that our data did not reach statistical significance because of the small sample size.

Our study has some limitations. First, due to the real-life cohort design of this study, the number of patients at each visit varied. Second, this is a retrospective study with a reduced sample size. Third, the results are from a single center. Therefore, these limitations should be considered when interpreting the results.

## Conclusion

In our real-life cohort, most patients with PH treated with riociguat showed stable or improved risk parameters, especially the 6MWT, at 3 years of follow-up. Moreover, our data was able to reproduce the results of pivotal studies during our follow-up.
